# Postoperative morbidity after adenotonsillectomy versus adenopharyngoplasty in young children with obstructive sleep apnea: an RCT

**DOI:** 10.1007/s00405-020-06035-2

**Published:** 2020-05-16

**Authors:** Johan Fehrm, Anna Borgström, Pia Nerfeldt, Danielle Friberg

**Affiliations:** 1 Department of Otorhinolaryngology, Karolinska University Hospital, Stockholm, Sweden; 2 Department of Clinical Science, Intervention and Technology, CLINTEC, Karolinska Institutet, Stockholm, Sweden; 3 Division of Otorhinolaryngology, Department of Surgical Science, Uppsala University, Uppsala, Sweden

**Keywords:** Obstructive sleep apnea, Adenotonsillectomy, Adenopharyngoplasty, Pharyngoplasty, Tonsillar pillar closure, Tonsillectomy

## Abstract

**Purpose:**

In our previous randomized controlled trial (RCT), comparing adenotonsillectomy (ATE) with adenopharyngoplasty (APP) in children with severe obstructive sleep apnea (OSA), there were no differences in respiratory sleep parameters or quality of life. The purpose of the present report was to evaluate postoperative morbidity from this RCT.

**Methods:**

The study was a blinded RCT in 83 children (ATE = 47; APP = 36), 2–4 years of age, with an obstructive apnea–hypopnea index of ≥ 10. Pain was assessed from the first until the tenth day after surgery with a logbook that reported pain by child (FPS-R, Faces Pain Scale-Revised) and caregiver (visual analogue scale), analgesic use, return to normal diet, and weight change. Bleeding, infection, satisfaction with treatment, speech, and swallowing were assessed with a questionnaire and medical records 6 months after surgery.

**Results:**

Sixty-four children (77%) returned the logbook and 65 (78%) answered the questionnaire. The median (interquartile range) day the children graded themselves as pain free (FPS-R = 0) was 7 (6–10) after ATE, compared with 9 (7 to > 10) after APP (*p* = 0.018). There were no other significant differences between the groups regarding any other pain-related outcomes, bleeding, infection, satisfaction, swallowing, or speech, but three children (11%) reported impaired speech after APP compared to none after ATE (*p* = 0.067).

**Conclusion:**

The results regarding postoperative morbidity were in favor of ATE and the results from our previous report showed no advantages of APP. Therefore, APP should not be recommended in young, otherwise healthy children with OSA.

## Introduction

Adenotonsillectomy (ATE) is a common procedure and the primary treatment for children with obstructive sleep apnea (OSA) [1, 2]. Previous studies have shown ATE to be an effective treatment to improve quality of life, respiratory sleep parameters, and behavior [3]. Even so, persistent OSA has been reported to be 13–75% after ATE [4–8], and alternative surgical treatment methods have been proposed to improve the results. For instance, studies have indicated that ATE with closure of the tonsillar pillars, referred to as adenopharyngoplasty (APP), has been more effective for improving the obstructive apnea–hypopnea index (OAHI) [9–11]. However, a recent randomized controlled trial (RCT) by our research group in otherwise healthy children (*n* = 83), 2–4 years of age, with severe OSA (OAHI ≥ 10) did not show that APP was more effective than ATE in improving respiratory sleep parameters or quality of life after 6 months [12].

Even so, APP might have other benefits. Covering the tonsillar fossa after ATE could reduce the pain and the risk for postoperative bleeding, but the results from previous studies are not consistent. For instance, Senska et al. [13] showed in a retrospective study (*n* = 2000) that the rate of postoperative bleeding was lower after tonsillar pillar closure, but an RCT (*n* = 763) by Matt et al. [14] did not show a reduced risk for postoperative bleeding. Further, an RCT (*n* = 39) by Genç et al. showed that covering of the tonsillar fossa after ATE reduced the postoperative pain, but two RCTs from Fornazieri et al. (*n* = 132) [15] and Friedman et al. (*n* = 60) [10] did not show reduced pain after tonsillar pillar closure. The previously mentioned large RCT by Matt et al. [14] even showed increased postoperative pain after tonsillar pillar closure.

Even though APP is not generally recommended for otherwise healthy children, it is an alternative treatment method that can be used in selected cases; therefore, it is important to evaluate risks and benefits associated with the procedure. The present study analyzed secondary outcomes from our previously mentioned RCT [12]. The aim was to evaluate postoperative morbidity (e.g. postoperative pain, bleeding, infection, satisfaction with treatment, and impaired speech and swallowing) after APP compared to ATE.

## Materials and methods

### Study design and population

This study analyzed data regarding postoperative morbidity from a blinded, prospective RCT comparing ATE with APP. The study was conducted at the Otorhinolaryngology Department at Karolinska University Hospital in Stockholm, Sweden, between December 2014 and November 2016. The original study was designed to evaluate the effect on respiratory sleep parameters, measured with polysomnography (PSG), and is described in more detail in the original article [12]. The power analysis was based on changes in OAHI and not on the secondary outcomes analyzed in this report.

The children included in the study were ≥ 2 to < 5 years of age and otherwise healthy, and had severe OSA (defined as OAHI ≥ 10), tonsil hypertrophy 2–4 (according to Brodsky [16]), and no bleeding disorders.

The children were randomized at the day of operation with sealed envelopes, in block of tens, and with an allocation ratio of 1:1. The researchers, PSG scorer, children, and caregivers were blinded to surgical method.

The study was approved by the Swedish Regional Ethics Board in Stockholm, Sweden (dnr 2014/1000-31/1).

### Intervention

All children were operated on with cold steel technique. The tonsils were removed by blunt extracapsular dissection, and the adenoid was removed with a ring knife. The children in the APP group also had their tonsillar pillars lateralized and closed. This was performed with two inverted sutures, Monocryl 4/0 (Ethicon, USA), on each side, including fibers of the palatopharyngeus muscle. All children received locally administrated bupivacaine perioperatively, and perioperative hemostasis was obtained with compression and bipolar diathermia. Perioperative blood loss was registered by the surgeon.

All children were prescribed analgesics, and the caregivers received a written schedule: ibuprofen 16–40 mg/kg/day, and paracetamol 80–100 mg/kg/day the first three days followed by 65–75 mg/kg/day. The caregivers were told to treat their children as long as they showed signs of pain. No antibiotics were given peri- or postoperatively.

### Outcomes

**Fig. 1:**
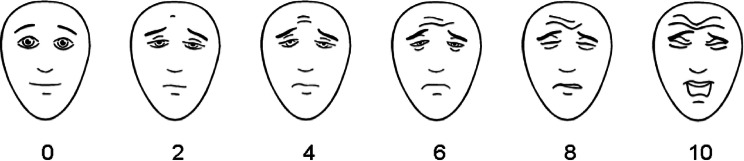
Faces Pain Scale-Revised (FPS-R). https://www.iasp-pain.org/fpsr. Copyright © 2001, International Association for the Study of Pain^®^. Reproduced with permission

All patients received a logbook where pain, analgesics given, and food intake were registered, from the first until the tenth day after surgery. Pain was assessed three times per day by both the children and the caregivers. The children used a standardized self-reporting scale (0–10) called the Faces Pain Scale-Revised (FPS-R) (Fig. 1). It consists of six different faces, is validated for children from 4 years of age, and is recommended by PedIMMPACT (Pediatric Initiative on Methods, Measurement, and Pain Assessment in Clinical Trials) [17–19]. The caregivers assessed the pain, from 1 to 10, using a visual analogue scale (VAS). The food intake was registered by the caregivers as amount (less than normal, normal, or more than normal) and texture (liquid, soft, or normal). Also, the weight in kilograms (kg) was registered, using the same scale, on the first and tenth day after surgery.

The data retrieved from the logbook were evaluated according to seven different pain-related outcomes: 1, first day when the child was pain free (FPS-R = 0); 2, first day when the child had FPS-R < 6; 3, first day when the caregiver estimated the child to be free of pain (VAS = 1); 4, first day when the caregiver estimated the child to have VAS ≤ 5; 5, first day without analgesics; 6, first day with normal diet (defined as normal texture in combination with normal or more than normal amount); and 7, mean weight change.

Postoperative bleeding and infection were assessed by evaluating clinical records and a questionnaire at the six-month follow-up. Only bleeding that required surgical treatment or readmission was defined as postoperative bleeding. Perioperative blood loss was also evaluated.

Further, postoperatively, the caregivers answered a questionnaire at the six-month follow-up regarding global satisfaction with treatment (yes or no), speech (improved, unchanged, worse, or much worse) and swallowing (improved, unchanged, worse, or much worse). Swallowing and speech were dichotomized to impaired (worse or much worse) and not impaired (improved or unchanged).

### Statistical analysis

The analysis was per protocol. The pain-related outcomes are reported as the median (interquartile range), and the group differences were analyzed with log-rank tests (nonparametric) and illustrated with Kaplan–Meier plots. The mean weight in kg and mean perioperative blood loss in ml are reported with standard deviations (SD) or 95% confidence intervals (CI), and were analyzed with independent *t*-tests (parametric). Postoperative bleeding, infection, global satisfaction with treatment, impaired speech, and impaired swallowing are reported as number (*n*) and percent (%), and were analyzed with Fisher’s exact test (nonparametric).

All data were analyzed with Stata 15 (StataCorp, USA).

## Results

**Fig. 2:**
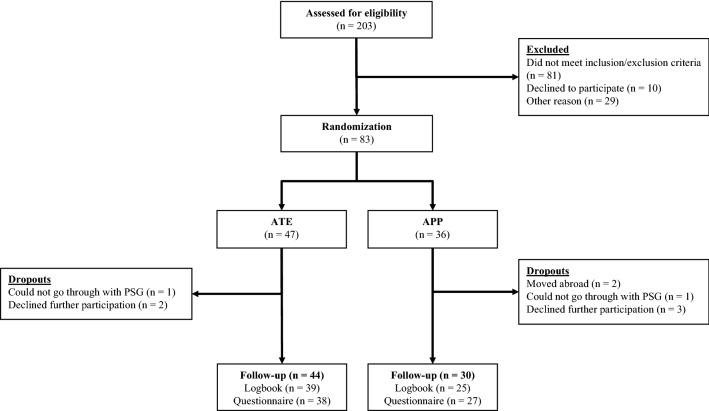
Flow of participants. *ATE* adenotonsillectomy, *APP* adenopharyngoplasty, *PSG* polysomnography

Eighty-three children were randomized to ATE (*n* = 47) or APP (*n* = 36); 64 (77%) returned the logbook (ATE, 39 [83%]; APP, 25 [69%]); and 65 (78%) answered the questionnaire regarding bleeding, infection, satisfaction with treatment, speech, and swallowing (ATE, 38 [81%]; APP, 27 [75%]) (Fig. 2). Data regarding peri- and postoperative bleeding, as well as postoperative infection, were obtained in all children through medical records.

The groups were similar at baseline (Table 1).

**Table 1:** Baseline characteristics

Characteristic	ATE (*n* = 47)	APP (*n* = 36)
Age at intervention, mean (SD), months	36.3 (9.7)	37.0 (8.7)
Male sex, no. (%)	26 (55)	23 (64)
Length, mean (SD), cm	93.2 (6.6)	93.5 (6.6)
Weight, mean (SD), kg	14.2 (2.6)	14.1 (2.8)
BMI z-score, mean (SD)	− 0.08 (1.46)	− 0.20 (1.52)
Tonsil size, ^a^median (IQR)	4 (3–4)	3.5 (3–4)
OAHI, mean (SD), events/hour of sleep	23.7 (11.5)	23.8 (11.5)

*ATE* adenotonsillectomy, *APP* adenopharyngoplasty, *n* number, *SD* standard deviation, *BMI* body mass index, *IQR* interquartile range, *OAHI* Obstructive Apnea–Hypopnea Index

^a^Tonsil size scored according to Brodsky

### Pain

**Fig. 3:**
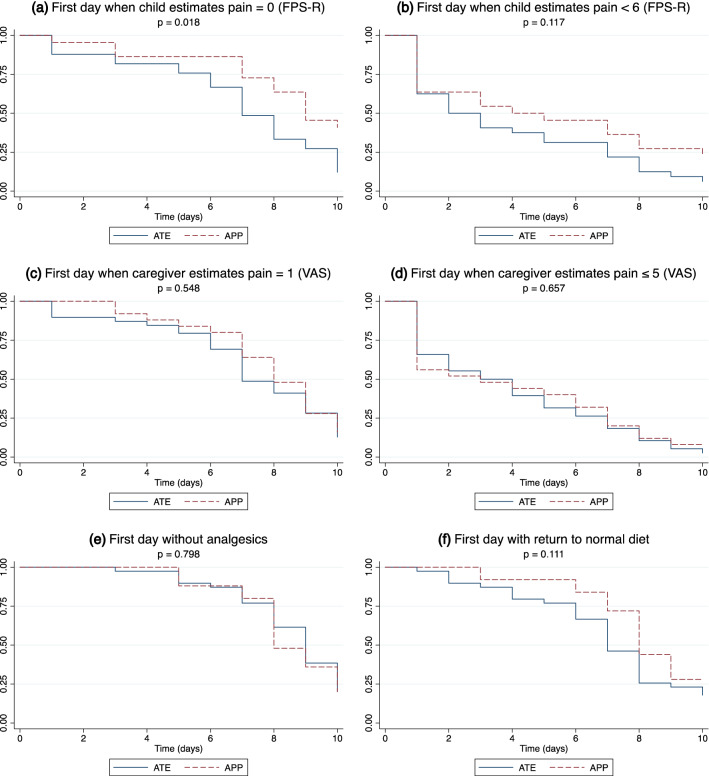
**a**–**f** Kaplan–Meier plots for six pain-related outcomes in both groups. *P*-values for group comparison with log-rank tests. *ATE* adenotonsillectomy, *APP* adenopharyngoplasty

The results of the seven pain-related outcomes are reported in Table 2, and six of them are illustrated in Fig. 3. There was a significant difference regarding the first day that the children graded themselves as pain free (FPS-R = 0). Median day (interquartile range) was 7 (6–10) in the ATE group, compared with 9 (7 to > 10) in the APP group (*p* = 0.018). There were no significant differences in mean weight change (− 0.2 kg; 95% CI − 0.5 to 0.1) or in any other pain-related outcomes.

**Table 2:** Pain-related outcomes for adenotonsillectomy versus adenopharyngoplasty

Parameter	*n*	ATE	*n*	APP	*p*
First day when child estimates pain = 0 (FPS-R)	33	7 (6–10)	22	9 (7 to > 10)	**0.018**
First day when child estimates pain < 6 (FPS-R)	32	2 (1–7)	22	4 (1–10)	0.117
First day when caregiver estimates pain = 1 (VAS)	39	7 (6–10)	25	8 (7–10)	0.548
First day when caregiver estimates pain ≤ 5 (VAS)	38	3 (1–7)	25	3 (1–7)	0.657
First day without analgesics	39	9 (8–10)	25	8 (8–10)	0.798
First day with return to normal diet	39	7 (6–9)	25	8 (7 to > 10)	0.111
Weight change (kg)	35	0.0 (0.6)	22	0.1 (0.5)	0.273

Data are expressed as median, with interquartile range, and the groups are compared with log-rank tests, except for weight change

The weight change is expressed as mean, with standard deviations, and the groups are compared with an independent *t* test

*ATE* adenotonsillectomy, *APP* adenopharyngoplasty, *FPS-R* Faces Pain Scale-Revised, *VAS* visual analogue scale, *kg* kilograms, *n* number of patients

### Bleeding and other postoperative outcomes

There were no significant differences in peri- and postoperative bleeding between the groups (Table 3). One patient in the ATE group was readmitted six days after surgery due to postoperative bleeding from the tonsillectomy, and one patient in the APP group was readmitted the day after surgery due to bleeding from the adenoidectomy. None of these patients needed surgical intervention.

**Table 3:** Bleeding and other postoperative outcomes for adenotonsillectomy versus adenopharyngoplasty

Parameter	*n*	ATE	*n*	APP	*p*^a^
Perioperative bleeding, ml (SD)	47	34 (17)	36	37 (21)	0.508
Postoperative bleeding, *n* (%)	47	1 (2)	36	1 (3)	1.000
Postoperative infection, *n* (%)	47	0 (0)	36	1 (3)	0.434
Satisfied with treatment, *n* (%)	36	36 (100)	24	23 (96)	0.400
Impaired swallowing, *n* (%)	36	1 (3)	24	0 (0)	1.000
Impaired speech, *n* (%)	38	0 (0)	27	3 (11)	0.067

*ATE* adenotonsillectomy, *APP* adenopharyngoplasty, *n* number, *SD* standard deviation, *ml* milliliter

^a^The groups were compared with Fisher’s exact test, except for the mean perioperative bleeding (ml), which was analyzed with an independent *t* test

One patient in the APP group was diagnosed with a postoperative infection nine days after surgery and was then treated with phenoxymethylpenicillin.

There were no significant differences between the groups in satisfaction with treatment, speech, and swallowing; however, three children in the APP group reported worse speech (none responded much worse) compared to none in the ATE group (*p* = 0.067). One child reported much worse swallowing after ATE, while no children had problems with swallowing after APP (*p* = 1.000) (Table 3).

## Discussion

This study analyzed secondary outcomes from an RCT (*n* = 83) comparing ATE with APP in otherwise healthy children, 2–4 years of age, with severe OSA. The original RCT did not show that APP was superior to ATE in improving respiratory PSG parameters or quality of life after 6 months [12]. The results from the present study also suggest that there were no advantages of APP compared to ATE regarding postoperative pain, bleeding, infection, satisfaction with treatment, speech, or swallowing.

The children scored themselves as pain free (FPS-R = 0) seven days after ATE, compared to nine days after APP, and the difference was significant. The result after ATE was similar in an RCT (*n* = 79, 2–6 years of age) by Borgstrom et al. [20], where the children scored themselves as pain free after 8 days. Even though the FPS-R is useful to evaluate pain after ATE [21], it is not validated or recommended for children less than four years of age [19]. In the present study, only 13 (20%) of the children who answered the logbook were four years old and therefore these results have to be interpreted with caution.

There were no significant differences regarding pain as reported by the caregivers (VAS), number of days with analgesics, when the children returned to normal diet, or any changes in weight. These outcomes, in combination, indicate that there were no differences in postoperative pain between ATE and APP. Previous studies have shown varied results in postoperative pain, and it can be difficult to compare the results, due to different study designs, methods of evaluating pain, statistical analyses, and the children’s ages. For instance, Matt et al. showed in an RCT (*n* = 763, 8–264 months of age) increased pain after APP compared to ATE, but in that study the children were their own controls, meaning one side was left open and the other was closed following ATE. Evaluating pain between two methods in the same patient has its limitations, especially in younger children, where it can be hard to distinguish between the two sides.

However, the results from the present study are consistent with findings in two other RCTs with similar study design, techniques, and outcomes. Friedman et al. (*n* = 60) [10] did not show a difference between the groups in return to normal diet, and Fornazieri et al. (*n* = 132) [15] did not show a difference between in self-reported pain (using a faces pain scale) or return to normal diet.

There were no significant differences in peri- or postoperative bleeding, and only one patient in each group (ATE, 2%; APP, 3%) had to be readmitted for postoperative bleeding. Previous studies have shown contradictory results regarding postoperative bleeding, but like the present study, many suffer from a small sample size. There are, however, two large studies by Matt et al. (*n* = 763) [14] and Senska et al. (*n* = 2000) [13]. In the RCT by Matt et al., there was no difference in postoperative bleeding, but in the retrospective study by Senska, the need for second surgery due to bleeding was almost halved, in favor of APP. There are several factors that might explain these differences, such as study design, surgical technique (e.g. how the mucosa and muscle were used to suture the tonsillar pillars), and choice of suture type.

There were no significant differences in postoperative infection rates, which is consistent with previous studies [15, 22], and both groups were satisfied with the given treatment.

Swallowing and speech disorders have not been previously well documented after APP in children, but surgical treatment in adults, for example, uvulopalatopharyngoplasty, is known to affect speech and swallowing [23]. In the present study there were no significant differences regarding impaired speech or swallowing, but the study was not powered for these outcomes, and notably, there were three children who reported impaired speech after APP compared to none after ATE. Even though there was no significant difference (*p* = 0.067), the result in this small sample indicates that there might be impaired speech after APP, and further studies with validated methods are needed to evaluate this.

These results, combined with the results from the previous report [12], did not show any differences between APP and ATE regarding postoperative morbidity, improving PSG parameters or quality of life. However, APP is a more extended method and it slightly increases the operating time by 8 min [13]. Altogether, APP showed no advantages compared to ATE, and therefore, ATE should still be considered as the primary treatment method for otherwise healthy children with severe OSA.

The major strength of this study is the randomized study design, where caregivers, children, and researchers were blinded for treatment allocation. Also, there was a low dropout rate, and the children were between two and four years of age. There are few studies in these young children, even though it is a common group to receive surgery for OSA.

There are several limitations in this study, primarily that the sample size is small and that the power might not be sufficient for these secondary outcomes. Further, the FPS-R is validated for children from four years of age, and a majority of the children in the present study were younger. However, it is difficult to assess self-reported pain in young children, and the FPS-R was used in the absence of other validated methods.

Also, speech and swallowing were evaluated with a single question answered by the caregiver, and not by any validated methods, but it is difficult to objectively measure these parameters in young children.

## Conclusion

There was a significant difference in one of the self-reported pain parameters in favor of ATE, but the FPS-R is difficult to assess in young children. There were no other significant differences, and the results from this RCT suggest that there were no advantages of APP compared to ATE regarding postoperative pain, bleeding, infection, satisfaction with treatment, speech, and swallowing. Also, in our previous report there were no advantages of APP in improving respiratory sleep parameters or quality of life. Therefore, APP should not be recommended as treatment for young, otherwise healthy children with OSA However, this was a small study and further studies are needed to confirm these results.
